# Assessing accuracy of an electronic provincial medication repository

**DOI:** 10.1186/1472-6947-12-42

**Published:** 2012-05-23

**Authors:** Morgan Price, Mike Bowen, Francis Lau, Nicole Kitson, Stan Bardal

**Affiliations:** 1 University of British Columbia, Vancouver, Canada; 2 University of Victoria, Finnerty Road Victoria, British Columbia, V8W 2Y2, Canada

## Abstract

**Background:**

Jurisdictional drug information systems are being implemented in many regions around the world. British Columbia, Canada has had a provincial medication dispensing record, PharmaNet, system since 1995. Little is known about how accurately PharmaNet reflects actual medication usage.

**Methods:**

This prospective, multi-centre study compared pharmacist collected Best Possible Medication Histories (BPMH) to PharmaNet profiles to assess accuracy of the PharmaNet profiles for patients receiving a BPMH as part of clinical care. A review panel examined the anonymized BPMHs and discrepancies to estimate clinical significance of discrepancies.

**Results:**

16% of medication profiles were accurate, with 48% of the discrepant profiles considered potentially clinically significant by the clinical review panel. Cardiac medications tended to be more accurate (e.g. ramipril was accurate >90% of the time), while insulin, warfarin, salbutamol and pain relief medications were often inaccurate (80–85% of the time). 1215 sequential BPMHs were collected and reviewed for this study.

**Conclusions:**

The PharmaNet medication repository has a low accuracy and should be used in conjunction with other sources for medication histories for clinical or research purposes. This finding is consistent with other, smaller medication repository accuracy studies in other jurisdictions. Our study highlights specific medications that tend to be lower in accuracy.

## Background

Medication errors are a common source of harm or potential harm to patients [1,2]. Types of medication errors include: medications inadvertently stopped (omission) or restarted (commission), unwanted dosage or frequency changes, and unintended drug interactions [3,4]. In a review by Bates, prescription medication errors occurred in up to 67% of patients being admitted to hospital with 11–59% of those being clinically important [5,6]. Poor medication histories are a common source of medication error [7] and better history taking, communication, and electronic tools to support medication reconciliation, such as community pharmacy databases are means by which such errors can be reduced [2,8-12]. However, there may be unintended consequences of using Information Communication Technologies [13,14].

PharmaNet is British Columbia’s (BC) provincial repository of medication dispensings from community pharmacies. Established in 1995, it connects all community pharmacies with the goal “to improve prescription safety and support prescription claim processing” [15]. In 2007, it processed over 47 million prescription dispensings. Authorized clinicians in community pharmacies, hospitals, emergency departments and medical practices can access PharmaNet to view medication dispensing records. Its use is widespread among pharmacists in BC as it is also the mechanism for billing for community dispensings. A survey of BC physicians reported that there was a perceived impact on prescribing practice in 20% of treatment decisions [16].A study of 55 admission medication histories showed reviewing PharmaNet helped identify drug therapy problems but did not appear to improve medication histories [17].

PharmaNet has been used to answer research questions about prescription rates and adherence in BC [18,19]. These studies assume the data in PharmaNet is an accurate reflection of the medications being taken. While a prospective trial on adherence to medications in 43 congestive heart failure patients showed PharmaNet to have “moderate to high” correlation to the comparative medication event monitoring system [20], a 20 patient study in 2005 compared pharmacist taken Best Possible Medication Histories (BPMHs) to PharmaNet profiles and discovered 60% of patients had inaccurate PharmaNet profiles [21]. PharmaNet was designed to record the dispensing record of medications and may not reflect usage of that medication. Other ambulatory care electronic medical records have been shown to have inaccurate medication lists [22,23]. Thus, clinicians may turn to regional repositories as a source of truth. They have been promoted to improve medication safety, one of the higher sources of medication error. However, small studies in other jurisdictions have also found low accuracy rates of jurisdictional medication repositories [20] suggesting that there is a real accuracy issue and that it may not be unique to PharmaNet and thus further work is needed in quantifying the gaps before changes are made to improve accuracy and usability of these systems. There may be unintended consequences if clinicians and researchers assume jurisdictional medication records are accurate and they are not.

To explore this issue, we assessed the accuracy of BC’s PharmaNet application, a well established jurisdictional dispensing repository.

1. How accurate is a jurisdictional dispensing record, such as PharmaNet, compared to BPMHs for patients who receive BPMH as part of routine care?

2. What types of discrepancies are most common in a jurisdictional repository?

3. What medications are most accurate and which are frequently discrepant?

4. What is the rate of potentially significant discrepancies amongst all discrepancies?

## Methods

### Study participants

This six-month, multi-centre, prospective study engaged pharmacists to collect sequential patient medication profiles. Joint University of Victoria and Vancouver Island Health Authority ethics approval was received (#J2010-104, 2010). Pharmacists were recruited through information sessions at hospital pharmacy rounds and then trained for the study. Patient eligibility criteria included having clinical need for a BPMH and a recruited pharmacist having access to the PharmaNet profile at the time of the BPMH (terminals were readily available at all sites). All patients who received a BPMH by a recruited pharmacist during the study were included in our analysis.

Pharmacists documented the patient’s BPMH in a Microsoft Access database as part of routine care and were also asked to document any discrepancies discovered between their BPMH and the patient’s PharmaNet profile.^a^ Discrepancy types were predefined: medication missing on PharmaNet profile, medication erroneously flagged as “current” on profile but not being taken, medication missing the “current” flag on PharmaNet Profile (NOTE: the current flag was an additional feature provided in the vendor software used to display PharmaNet profiles), error in dose, error in route, error in frequency, and unclear ingredient or instruction.

### Data extraction

Anonymized data was extracted from the pharmacist’s BPMH electronic repository by one of the pharmacists and provided to the researchers electronically as an excel file. Data included: age, gender, current medication list, medication discrepancies, and any comments provided by the pharmacist pertaining to the documented medication discrepancies.

### Data analysis

Characteristics of the anonymized profiles and of the individual medication entries were tabulated. Descriptive statistics were used in summarizing the data. For each discrepancy type (from Bates et al.) [5], the overall number of discrepant medication entries was tabulated. Medications were grouped by Anatomic Therapeutic Class (ATC). Most accurate and most discrepant medication classes were then identified.

A Clinical Study Panel was established with a clinical pharmacist, a pharmacologist, and a family physician to review a random sample of anonymized profiles with discrepancies to estimate the potential risk of a significant Adverse Drug Event (ADE). The Panel reviewed each profile and estimated risk for potential ADE if all medications were prescribed *with* the noted discrepancies. A Delphi approach was used [24,25], modified for this study. Specifically, each panel member individually assessed the risk of a potentially significant ADE. A potentially significant ADE was defined as: requiring admission, increasing risk of serious complication, deterioration of clinical condition (e.g. infection, cancer), or worsening chronic illness (e.g. depression) that could impact daily function within a two month period. This definition is consistent with previously described scales [26].

A random sample of the patient profiles with discrepancies was generated from within Microsoft Access to ensure a margin of error of less than 5% at a 95% Confidence Interval. Where consensus could not be reached, an adjudicator (author, M.P.) reviewed the cases, the rationale for individual scores from the Panel, additional evidence, and made a decision on whether or not the discrepancies were potentially significant.

Profiles were grouped according to the number of medication discrepancies they contained. For each group, the number and rate of potentially significant ADEs was tabulated and the medications most frequently associated with potentially significant ADEs were identified.

## Results

21 pharmacists from two hospitals were recruited to the study. Pharmacists performed BPMHs at three locations within the hospitals: Emergency Department, inpatient wards, and pre-admission clinics as part of routine care. Data was collected from January-May 2011. 1215 anonymized patient medication profiles were sequentially collected, containing 7791 medications. The average patient age was 65.8 years, and 51.9% of patients were female. There was an average of 6.4 medications per profile. 1024/1215 profiles (84.3%) were found to contain at least one discrepancy. On average, discrepant profiles contained 3.2 discrepancies. 3256/7791 (41.8%) of medications exhibited discrepancies (Table 1). Of the 1024 total discrepant profiles, 84 were discrepant solely due to a current flag related error. If these errors were removed (as this specific feature was not part of the PharmaNet specification), the overall rate of discrepant profiles would fall from 84.3% to 77.4% (940/1215).

**Table 1:** Summary of anonymized medication profiles collected for this study

Study Sites	2 hospitals
Participating Pharmacists	21
Number of anonymized profiles (male/female)	1215 (584/631)
Mean Age (range)	Mean 65.8 +/− 14.8 yrs. (Min 1, Max 98)
Total Medications entries documented	7791
Medications per patient	6.4
Medications with at least one discrepancy (%)	3256 (41.8%)
Profiles with at least one discrepancy (%)	1024 (84.3%)
Average # of discrepancies/profile (95% CI)	3.2 (3.04-3.36)

*Medication missing from the Profile* was the most frequently documented discrepancy type (45%). Medications that could be purchased over the counter accounted for the majority of these medication discrepancies (up to 82%, although many of these would be recommended by prescribers but purchased over the counter). *Medication listed on the Profile, but missing the “current” flag* was the second most commonly documented discrepancy with insulin, salbutamol and codeine being most frequently discrepant in this manner (26%). Discrepancies in dose or frequency accounted for a further 25.05% of all recorded discrepancies. Table 2 summarizes discrepancies by type. Appendix A provides a detailed list of the five most frequently discrepant medication classes for each discrepancy type.

**Table 2:** Discrepancies found in medications within PharmaNet profiles as compared to BPMH by type

**Discrepancy type**	**Number of medication discrepancies documented, by type**
Medication missing	1587 (44.78%)
Medication missing “current” flag indicator	920 (25.96%)
Medication listed with inaccurate frequency	520 (14.67%)
Medication listed with inaccurate dose	368 (10.38%)
Discontinued medication listed as “current”	108 (3.05%)
Unclear ingredient information or instructions	39 (1.10%)
Medication listed with inaccurate route	2 (0.05%)
Total Number of Medication Discrepancies	3544 (100%)

Table 3 lists the top five most accurate and five most discrepant medication classes that were frequently dispensed (i.e. used by more than 5% of study patients). Ramipril was the most accurate medication in PharmaNet with discrepancies occurring in only 18/207 (8.7%) appearances. Not surprisingly, artificial tears and ibuprofen, acetaminophen and acetylsalicylic acid were among the most discrepant, largely because as over the counter medications they rarely were captured in PharmaNet unless dispensed by a pharmacist. Of prescription medications, insulin frequently exhibited discrepancies (88.6%), with discrepancies of dose and missing current flags being most frequent. Warfarin’s high discrepancy rate (82.5%) was largely due to dose and frequency errors. Codeine & codeine combinations were routinely found to be discrepant (78.4%) largely due to discrepancies in frequency and missing current flags. Opiate agonists of all types had discrepancies in 46.1% of entries (159/345) with discrepancies of frequency and missing current flags being most common.

**Table 3:** Five best and worst documented medications in PharmaNet, determined by rate of discrepancy and being prescribed in more than 5% of patients (>60 patients)

		**Discrepancy Rate (discrepancies/total times documented)**
**Most accurate medication classes in PharmaNet**	Ramipril	8.70% (18/207)
	Hydrochlorothiazide	10.10% (21/208)
	Atorvastatin	10.50% (23/219)
	Levothyroxine Sodium	10.58% (22/208)
	Citalopram	10.61% (7/66)
**Most discrepant medication classes in PharmaNet**	Artificial Tears and Other Indifferent Preparations	98.44% (63/64)
	Insulin	88.57% (93/105)
	Ibuprofen, Acetaminophen and Acetylsalicylic Acid & combinations	86.07% (624/725)
	Warfarin	82.46% (94/114)
	Codeine & combinations	78.43% (80/102)

A collection of medications including vitamins, creams and other supplements (e.g. multivitamins, psyllium and calcium carbonate) were documented more than any other (427 times). Discrepancies were noted in 77.3% of entries, and were largely due to the medications being absent from the profile, or in the case of creams, containing ingredients that were not documented in PharmaNet.

The Panel reviewed 660/1024 anonymized medication profiles with discrepancies (2.28% margin of error @ 95% confidence interval). Consensus was reached on 630 profiles (95.4%). 30 required arbitration. Potentially significant ADEs occurred in 48.2% of reviewed cases (318/660), and more frequently in patients with multiple medications (Table 4). Discrepant medications most commonly implicated in potentially serious cases included: warfarin, salbutamol, insulin, codeine and combinations, clonazepam and prednisone.

**Table 4:** Number of profiles considered to have a potentially serious ADE as determined by the clinical study panel, summarized by number of discrepancies/profile

**Number of Profile discrepancies identified per BPMH**	**Number of Profiles (%)**	**Number of reviewed profiles considered to have a serious discrepancy**
0	191 (15.72%)	n/a
1	278 (22.88%)	40/174 (22.99%)
2-3	417 (34.32%)	128/279 (45.88%)
4-5	192 (15.80%)	75/119 (63.02%)
6-9	108 (8.89%)	60/72 (83.33%)
10+	29 (2.39%)	15/16 (93.75%)
Total	1215 (100%)	318/660 (48.18%)

## Discussion

This study revealed a high number of discrepancies (84.3%) between the gold standard Best Possible Medication History and medication profiles found in the PharmaNet provincial medication database. Several of these discrepancies were expected, based on the scope of PharmaNet (e.g. absent over the counter, HIV, and hospital dispensed medications); however, many were not. The most common error was missing medications, followed by medications that did not appear as active (or “Current”) on the PharmaNet profile although they were still being used. Almost half of profiles contained discrepancies that were determined to be potentially clinically significant if not corrected (318/660). That is, if the PharmaNet profile was considered accurate and was solely used to direct prescribing, there was a significant risk for medication errors that could lead to ADEs. There are implications based on the findings in this study that relate to use of PharmaNet for clinicians, researchers and the PharmaNet program.

PharmaNet should be used by clinicians in conjunction with other clinical information (such as a patient history), clinical judgment, and follow up to prevent potential ADEs. The risk of significant error increases with the number of medications a patient is taking. Clinicians should focus attention on the common medication classes listed in Table 5 due to the frequency with which they are discrepant or absent from the PharmaNet profile. Common and accurately recorded medications (~90% accuracy in PharmaNet) tended to be cardiovascular drugs (with the exclusion of ASA, 15% accuracy), thyroid medication, and citalopram (Table 3).

**Table 5:** Top suggestions for clinicians when using PharmaNet in conjunction with a medication history

**Medication**	**Discrepancy Rate**	**Suggestions**
Artificial tears	98.44% (63/64)	Be sure to ask about these over the counter medications as they are very rarely found on the Profile.
Insulin	88.57% (93/105)	Be sure to confirm the dose. In 30% of Profiles containing Insulin’s the dose being used will differ from that listed on PharmaNet.
		Likewise, the “Current” flag will not appear in 30% of Insulin containing Profiles.
Ibuprofen, Acetaminophen and Acetylsalicylic acid including combinations	86.07% (624/725)	Be sure to ask about these over the counter medications as they are very rarely found on the Profile.
Warfarin	82.46% (94/114)	Expect a dose and/or frequency discrepancy in nearly 60% of PharmaNet Profiles containing Warfarin.
Codeine & Codeine Combinations, Opiate agonists in general	78.43% (80/102), 46.09% (159/345)	Do not rely on the presence of the “Current” flag to detect Codeine and Opiate Agonists on the Profile; it is missing in 30% and 20% of cases, respectively.
		Discrepancies in frequency are equally as common.
Vitamins, creams and other supplements	77.28% (330/427)	Be sure to ask about vitamins and supplements as they are very rarely found on in PharmaNet.
		In the case of compounded creams, it may be necessary to contact the compounding pharmacy in order to deduce the ingredients as they are often not provided in PharmaNet.
Salbutamol & Related Drugs	68.15% (92/135)	Do not rely on the presence of the “Current” flag to detect Salbutamol and related drugs on the Profile; it is missing in 33% of cases.
		Discrepancies in frequency occur in 17% of cases.

For researchers using PharmaNet and potentially other, similar medication repository data, they should be aware of these limitations and not assume that PharmaNet accurately represents medication usage in a given population. Studies have assessed expected completeness of drug profiles, for example in Ontario [27]. However, researchers should also assess accuracy of instructions (if relevant) as these were also frequently inaccurate. This study highlights that many patients are not taking the medications as they were dispensed. This study has shown several medications are often absent from the profile (e.g. over the counter medications), and many are used differently than was recorded at the time of dispensing (e.g. dose and frequency discrepancies in warfarin, insulin, opiate agonists). When employing PharmaNet data to study medication usage, it would be important to consider validating in PharmaNet the accuracy of medications of interest.

For the PharmaNet program, which is undergoing enhancement [28], this study suggests that there may be value in allowing trained clinicians (e.g. physicians, pharmacists, nurse practitioners) to update patient profiles in order to correct medication data when medication changes are made or discrepancies are found (e.g. while performing a BPMH). A current or regular medication list is not available in the current PharmaNet design, but could be considered for future updates. As many of the discrepancies were medications that appeared expired that were in fact not expired, the concept of *clinicians* flagging medications as “current” or “ongoing” may be clinically relevant. Enhancements such as these would increase the reliability of PharmaNet and thus improve the efficiency of medication reconciliation during a BPMH.

Findings here were similar to a smaller study performed in Saskatchewan, where 39/50 (78%) of patients had at least one discrepancy on their profile [20]. Also, a study of 493 patients at Veteran Affairs found that their integrated primary care medication record was accurate in only 5.3% of patients [22], compared to a medication history. This suggests that the findings are not unique to BC and PharmaNet and other regional and jurisdictional repositories may have similar accuracy rates.

This study has added to our knowledge of accuracy of medication repositories by quantifying discrepancies in a large, prospective study of patients whom are typically at risk for medication errors. It has been able to describe, by medication class, some of the more common discrepancies as well as those medications that are accurately described in PharmaNet. Other jurisdictions have or are developing regionalized or national medication records such as in Australia, the US [29] and the NHS. Where these may rely on dispensing or prescribing information to populate their repositories, similar accuracy issues may exist.

### Limitations

There are several limitations to this study. First, this was not a randomized study of patients in BC; thus, the findings in this study may not be applicable to the entire population of the province. This patient population is likely more complex and on more medications than average. This study was completed within in BC with a single medication repository (PharmaNet). Medication profiles and discrepancy rates may be different in other parts of the world and where other features are incorporated into the tools (e.g. reconciliation). However, other studies suggest that there are significant error rates in other repositories [22,23]. We did not perform chart reviews when estimating the potential impact of the observed discrepancies. Having a greater understanding of the patients’ medical history would have allowed the clinical study panel to be more precise in their estimations of severity of potential ADEs. This would not have allowed for the anonymous data collection and would have limited the number of cases that were reviewed. It is not typical that providers would rely solely on the PharmaNet profile for prescribing without follow up, and patients would likely be aware if medications missing or their symptoms were not being managed. Thus, the construct for the Panel assessment, while useful in this study, is somewhat theoretical.

### Future work

This study was designed as a baseline study to assess the current version of PharmaNet. PharmaNet is being updated, with new features to be deployed in the coming years. A repeat of this study once an enhanced PharmaNet is deployed and stabilized would provide evidence to the impact of changes in the design of PharmaNet. Comparative studies could be completed in other medication repositories. Further studies could assess the rates of medication errors and potential ADEs, comparing patients who receive BPMH on admission/discharge to hospital and those who do not (i.e. those who rely more on the PharmaNet profile). Controlled studies should be performed that look at the addition and use of specific features that support medication reconciliation. These could discover what functions are needed (both social and technical) to improve accuracy of the repositories.

## Conclusions

This study examined the accuracy of a provincial medication repository by comparing the electronic medication profile to pharmacist collected best possible medication histories in patients who were deemed in need of a BPMH. A significant number of these patients (84%) had at least one discrepancy. 48% (2.28% error; 95% CI) of those patients had discrepancies that were deemed potentially clinically significant. This study suggests that using the electronic medication profile alone is insufficient for completing a medication history and must be reviewed in the context of other elements of history from the patient and other health records. The PharmaNet system was approximately 90% accurate in describing ramipril, hydrochlorothiazide, atorvastatin, levothyroxine sodium, and citalopram. Thus, could be used effectively to help streamline the reconciliation process for those medications. A medication history, supported by PharmaNet should focus on confirming the following medication classes: insulin, warfarin, codeine, salbutamol and ibuprofen/acetaminophen/acetylsalicylic acid, which were discrepant in 80–85% of cases. As expressed in a PharmaNet bulletin, “PharmaNet is not intended as a substitute for professional judgment. Information on PharmaNet is not exhaustive and cannot be relied upon as complete” [30].

## 

Top five medication discrepancies by discrepancy type, summarized by ATC class*

**Appendix A:** Top five medication discrepancies by discrepancy type, summarized by ATC class*

**Medications by ATC Class**	**Number of discrepancies**
**Medication missing**	
Acetylsalicylic Acid & Combinations	311
Uncoded vitamins, creams and other supplements	293
Acetaminophen & Combinations	206
Colecalciferol	92
Ibuprofen & Combinations	73
**Medication missing “current” flag indicator**	
Salbutamol	45
Codeine & Combinations	32
Insulins	31
Zopiclone	26
Lorazepam	23
**Medication listed with inaccurate frequency**	
Codeine & Combinations	32
Lorazepam	25
Salbutamol	23
Warfarin	22
Zopiclone	21
Hydromorphone	21
Medication listed with inaccurate dose	
Warfarin	44
Insulins	34
Zopiclone	18
Prednisone	14
Metformin	11
**Discontinued medication listed as “current”**	
Zopiclone	5
Amitriptyline	4
Uncoded vitamins, creams and other supplements	4
Raberprazole	3
Warfarin	3
Naproxen	3
Furosemide	3
Acetaminophen & Combinations	3
Clopidogrel	3
**Medication listed with unclear ingredient information or instructions**	
Warfarin	9
Uncoded vitamins, creams and other supplements	7
Cyanocobalamin	4
Hydrocortisone	3
Insulins	2
Diclofenac	2
Medication listed with inaccurate route	
Phenazopyridine	1
Uncoded vitamins, creams and other supplements	1

* The following ATC classes were combined for reporting purposes:

Acetaminophen & Combinations: ACETAMINOPHEN (PARACETAMOL) and ACETAMINOPHEN, COMB EXCL PSYCHOLEPTICS

Acetylsalicylic Acid & Combinations: ACETYLSALICYLIC ACID and ACETYLSALICYLIC ACID, COMB EXCL PSYCHOLEPTICS

Codeine & Combinations: CODEINE and CODEINE, COMBINATIONS EXCL. PSYCHOLEPTICS

Ibuprofen & Combinations: IBUPROFEN and IBUPROFEN, COMBINATIONS

Salbutamol: SALBUTAMOL and SALBUTAMOL AND OTHER DRUGS FOR OBSTRUCTIVE AIRWAY DISEASES

Insulins: INSULIN (HUMAN), INSULIN ASPART, INSULIN DETEMIR, INSULIN GLARGINE, INSULIN GLULISINE and INSULIN LISPRO

## Endnotes

^a^In BC pharmacists review PharmaNet as part of a BPMH.

## Competing interests

The authors declare that they have no conflict of interest.

## Authors’ contributions

MP was lead for the study, including initial design and primary author of the paper. MB was the research analyst on the study, designing data collection methods, completing data analysis and co-authoring the paper. FL was involved in the design of the study and in editing the paper. SB will a member of the clinical study panel and reviewed findings and edited the paper as a clinical pharmacologist. All authors read and appoved the final manuscript.

## Pre-publication history

The pre-publication history for this paper can be accessed here:

http://www.biomedcentral.com/1472-6947/12/42/prepub
